# Trends in Management and Outcomes of Blunt Thoracic Aortic Injury in the United States

**DOI:** 10.1016/j.ejvsvf.2025.07.004

**Published:** 2025-07-21

**Authors:** Anne-Sophie C. Romijn, Vinamr Rastogi, Patrick D. Conroy, Yuchen Liu, Sai Divya Yadavalli, Lars Stangenberg, Vincent Jongkind, Noelle N. Saillant, Hence J.M. Verhagen, Marc L. Schermerhorn

**Affiliations:** a Department of Surgery, Division of Trauma & Emergency Surgery, Massachusetts General Hospital, Harvard Medical School, Boston, MA, USA; b Department of Surgery, Division of Trauma & Emergency Surgery, Amsterdam University Medical Center, Amsterdam, the Netherlands; c Department of Surgery, Division of Vascular and Endovascular Surgery, Beth Israel Deaconess Medical Center, Harvard Medical School, Boston, MA, USA; d Department of Global Health and Population, Harvard T.H. Chan School of Public Health, Boston, MA, USA; e Department of Surgery, Division of Acute Care and Trauma Surgery, Boston Medical Center, Boston, MA, USA; f Department of Vascular Surgery, Erasmus University Medical Center, Rotterdam, the Netherlands; g Department of Surgery, Division of Vascular Surgery, Amsterdam University Medical Center, Amsterdam, the Netherlands; h Amsterdam Cardiovascular Sciences, Microcirculation, Amsterdam, the Netherlands

**Keywords:** Aortic trauma, Blunt thoracic aortic injury, Endovascular graft, Open repair, TEVAR

## Abstract

**Objective:**

Blunt thoracic aortic injury (BTAI) is a significant cause of mortality and morbidity in the United States. The diagnosis and treatment of BTAI has evolved over the last few decades. This study aimed to examine the incidence and outcomes of thoracic endovascular aortic repair (TEVAR), non-operative management (NOM), and open aortic repair (OAR) for BTAI over a 14 year period.

**Methods:**

Patients with BTAI from the National Inpatient Sample between 2006 – 2019 were studied and compared within three time periods: 2006–2010, 2011–2015, and 2016–2019. Waldtest and Pearson's chi-squared were performed to test whether management, patient characteristics, and in hospital mortality changed over time.

**Results:**

An estimate of 8 175 BTAI patients was identified, with an increasing estimate from 375 patients in 2006 to 750 patients in 2019. TEVAR utilisation increased from 17% to 37% during the study period, while those who received OAR and NOM decreased (OAR 16%–1.3%; NOM 67%–61%). There was an increasing trend in patients treated in an urban teaching hospital (2006–2010 *vs*. 2016–2019: 88% *vs*. 95%; *p* trend <0.001), and more patients were transferred from another hospital (12% vs. 18%; *p* trend = 0.027). Over the years, patients who received NOM were older (45 *vs*. 50 years; *p* < 0.001) and had more concurrent injuries, while the mortality rate among this group did not change (adjusted odds ratio [aOR] 0.65, 95% confidence interval [CI] 0.42–1.02; *p* = 0.060) Patients who received TEVAR were also older over the years (41 years *vs*. 46 years; *p* < 0.001), but they had fewer concurrent injuries, and the mortality rate remained stable (aOR 0.97, 95% CI 0.33–2.88; *p* = 0.96).

**Conclusion:**

The estimated number of patients with BTAI was twice as large in 2019 compared with 2006, and the use of TEVAR increased, largely replacing OAR.

## Highlights

• The incidence of blunt thoracic aortic injury (BTAI) doubled over the 14 year study period.
• The use of thoracic endovascular aortic repair (TEVAR) increased, largely replacing open aortic repair.
• Mortality rates within the BTAI cohort remained relatively stable over the years.
• There appears to be room for improvement in operative and conservative management.

## INTRODUCTION

Blunt thoracic aortic injury (BTAI) occurs due to rapid deceleration, causing tearing or avulsion of aortic tissue, most commonly caused by high speed crashes.^1^ BTAI is the second leading cause of death from blunt trauma after head injury and imposes a substantial disease burden in the United States (US) due to its high mortality and morbidity rates.^1^ It is estimated that the incidence of BTAI in patients suffering blunt thoracic trauma is 2%^2^ and pre-admission mortality has been demonstrated to be as high as 80% after BTAI.^3^^,^^4^

Diagnosis and treatment of BTAI have evolved in the last few decades. In 2005, the Food and Drug Administration (FDA) approved the use of thoracic endovascular aortic repair (TEVAR) for patients with aneurysms.^5^ After the feasibility of TEVAR in treating patients with BTAI was demonstrated by several studies, the use of TEVAR increased.^6^, ^7^, ^8^, ^9^ Concomitantly, the adoption of computed tomography angiography (CTA) as the imaging modality of choice enhanced the detection of smaller aortic injuries.^4^^,^^10^^,^^11^ Due to the increasing awareness of BTAI, a classification system based on the grading of the aortic wall injury visualised on CT or magnetic resonance imaging scans was proposed.^12^^,^^13^ In 2011, the Society of Vascular Surgery (SVS) generated guidelines for BTAI treatment, recommending non-operative management (NOM) for grade I aortic injury and TEVAR for grade II – IV aortic injury.^13^

It is unclear what the impact of these changes in practice has on the treatment and outcomes of patients with BTAI. This study aimed to investigate trends in the incidence and outcomes of patients with BTAI from 2006 – 2019, including TEVAR, NOM, and open aortic repair (OAR), evaluating in hospital outcomes and risk factors for mortality over the years.

## METHODS

### Data source

A retrospective cohort study of all hospitalisations for BTAI in the US from January 2006 to December 2019 was conducted using the National Inpatient Sample (NIS). Data were included up to 2019 because more recent years of the database were unavailable at the time of extraction. Although more recent data would have been preferable, the included data still allowed for meaningful and relevant analysis. This database is funded and maintained by the Healthcare Cost and Utilization Project and the Agency for Healthcare Research and Quality (AHRQ).^14^ The database is a compilation of 20% of all non-federal inpatient hospitalisations in the US, and actual annual US hospitalisation volumes are approximated with proper weighting procedures recommended by the AHRQ.^15^^,^^16^ The database covers more than 97% of the US population.^17^ The authors declare that all supporting data are available within the article. The present report adhered to the applicable STROBE (strengthening the reporting of observational studies in epidemiology) standards for observational studies.^18^ The Beth Israel Deaconess Medical Center Institutional Review Board approved this study and waived the need for written informed consent owing to the retrospective and de-identified nature of the NIS database.

### Patient cohort

All patients within the NIS diagnosed with BTAI between January 2006 and December 2019 were included. Patients were identified by using the ICD-9-CM and ICD-10-CM codes. The US began medically coding with the new ICD-10-CM PCS on 1 October 2015. Data from the first three quarters of 2015 were identified using ICD-9-CM, while data from the last quarter of 2015 were identified using ICD-10-CM. Hospitalised patients with an ICD-9-CM code containing 901.0 (injury to the thoracic aorta) or an ICD-10-CM code containing S250xxx (‘x’ denotes any number) were included. The following codes were included: S25.00 (unspecified injury of thoracic aorta), S25.01 (minor laceration of thoracic aorta), S25.02 (major laceration of thoracic aorta), S25.09 (other specified injury of thoracic aorta). ICD-9 and ICD-10 procedure codes were used to divide patients into the TEVAR and OAR cohorts. Patients with BTAI lacking a code for TEVAR or OAR were labelled NOM. All ICD-10-CM codes were independently reviewed for accuracy by multiple authors (A.R., V.R., and P.C.) and compared against previous studies.^19^ Any patients with concomitant penetrating trauma codes in any diagnosis code were excluded.

This study, examined the overall cohort of patients with BTAI and divided the patients into three groups based on the time periods: 2006–2010, 2011–2015, and 2016–2019 to evaluate trends over the years. This stratification was also applied separately to the three treatment modalities (TEVAR, NOM, and OAR).

### Variable and outcome definitions

Patient demographics, such as age, sex, race, and body mass index, were collected for all patients. Important comorbidity data were also collected, including hypertension, diabetes mellitus, congestive heart failure, chronic kidney disease, chronic obstructive pulmonary disease, and coronary artery disease, which were predefined Elixhauser covariates in the database.^19^ The NIS database does not contain data regarding the aortic injury grading, injury severity score (ISS), and abbreviated injury scale (AIS). Concomitant injuries and interventions during the same hospitalisation were collected. Intracranial interventions, thoracic interventions other than TEVAR (such as lung or heart procedures), and abdominal interventions were included for the concomitant interventions. Hospital characteristics included hospital setting (rural, urban non-teaching, and urban teaching) and hospital size, meaning the number of beds available in the hospital for patients (small, medium, and large).

The primary outcomes of the study were admissions, method of aortic injury repair, and in hospital death. The secondary outcomes were the trends of the proportions of each intervention method of BTAI over time, including NOM, TEVAR, and OAR, as well as the associated mortality for each method. Other secondary outcomes derived in this analysis were complications after each intervention method, including paraplegia, cardiac complications, pulmonary complications, post-operative acute renal failure, and post-operative sepsis.

### Statistical analysis

Continuous variables are presented as mean and standard deviation, since they had normal Gaussian distributions, and categorical variables are presented as weighted number of patients (percentages). Continuous variables were compared using the Wald test to assess for differences in the mean, and categorical variables were compared using the Pearson chi-square test. QQ plots were used to determine distribution. Linear regression analyses were conducted to determine the slope of the change in frequency by year. The Cochrane–Armitage trend test was also used for intervention method proportions and mortality across three distinct time cohorts (2006–2010, 2011–2015, and 2016–2019) to correct for yearly fluctuations. The primary endpoints were separately analysed for the overall cohort and the three intervention subgroups: OAR, TEVAR, and NOM.

A multivariable logistic regression was performed to identify factors associated with mortality in patients treated by NOM or TEVAR. Adjusted odds ratios (aORs) were calculated to quantify the independent association of each variable with mortality, while controlling for the effect of other variables in the model. Variables used in the multivariable model included: sex, non-white ethnicity, private insurer, traumatic brain injury (TBI), cardiac injury, lung injury, rib fracture, pneumothorax, haemothorax, haemopneumothorax, kidney injury, pelvic injury, spinal fracture with and without spinal cord ischaemia (SCI), intracranial intervention, thoracic intervention, abdominal intervention, and, in the TEVAR group, time to TEVAR. The inclusion of a variable was based on a combination of clinical relevance, prior evidence, and statistical considerations. All statistical analyses were performed using Stata, version 17.0 (StataCorp, College Station, TX, USA). All demographic variables had <5% missing values.

## RESULTS

### Overall BTAI cohort

**Figure 1:**
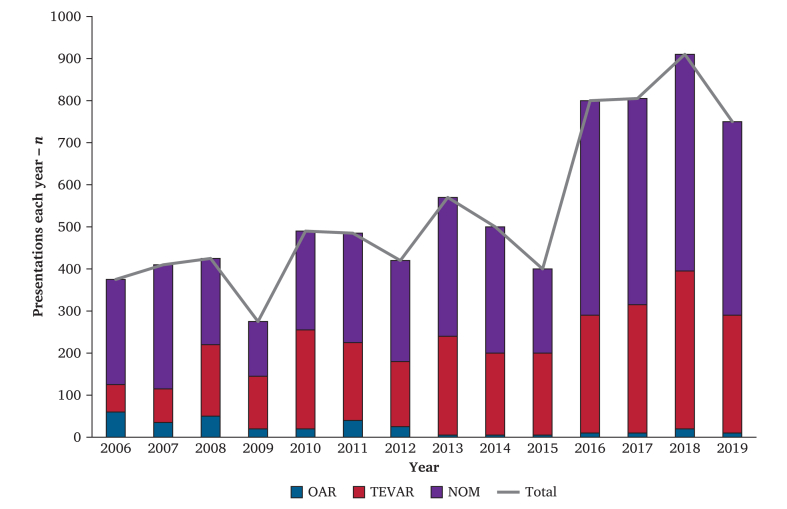
Presentation of all patients with blunt thoracic aortic injury and the three treatment modalities. NOM = non-operative management; OAR = open aortic repair; TEVAR = thoracic endovascular aortic repair.

**Figure 2:**
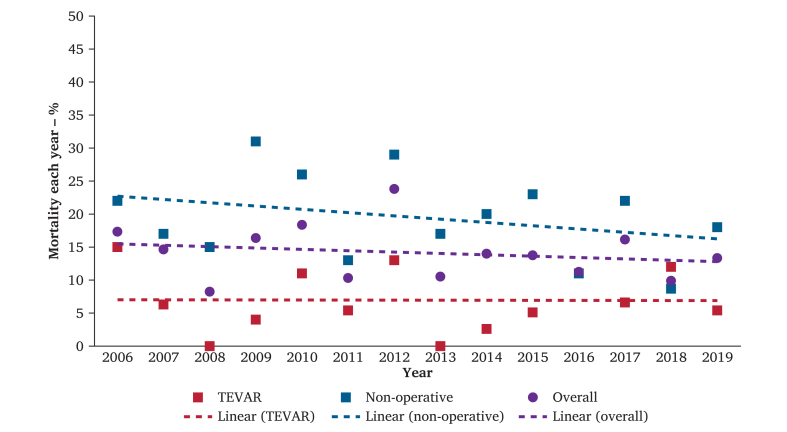
Linear regression of mortality in all blunt thoracic aortic injury patients, the thoracic endovascular aortic repair (TEVAR) cohort, and non-operative management cohort by year.

An estimated 8 175 BTAI patients were admitted to US hospitals from 2006 to 2019, of whom 2 890 (35%) were treated with TEVAR, 4 865 (60%) with NOM, and 420 (5.1%) with OAR. The annual number of patients who presented with BTAI increased per year from 375 patients in 2006 to 750 patients in 2019 (*p* < 0.001) (Fig. 1) (Supplementary Table S1). The proportion of patients treated with TEVAR increased from 17% in 2006 to 37% in 2019 (*p* = 0.13). The proportion of patients treated with OAR over the same period decreased from being the management in 16% of all BTAI patients in 2006 to 1.3% in 2019 (*p* < 0.001). The absolute number of patients who received NOM increased, while the proportion of patients treated with NOM decreased from 67% in 2006 to 61% in 2019 (*p* = 0.83). Fig. 2 demonstrates similar mortality rates per year for the overall cohort (*p* = 0.30), in the patients treated with TEVAR (*p* = 0.45), and patients receiving NOM (*p* = 0.12).

#### Patient characteristics

Evaluating the trend of the overall BTAI group in the three time periods (2006–2010, 2011–2015, 2016–2019), Table 1 shows that patients were statistically significantly older over the years (2006–2010 *vs*. 2016–2019; 44 years *vs*. 49 years; *p* trend <0.001). There was a statistically significant increasing trend in patients treated in an urban teaching hospital instead of urban non-teaching hospital or rural hospital (*p* trend <0.001). Also, more patients admitted to the hospital were transferred from another hospital (12% *vs*. 18%; *p* trend = 0.027).

**Table 1:** Demographics, comorbidities, hospital characteristics, concomitant injuries, interventions and outcomes in the BTAI cohort over time.

	All BTAI Patients						
	2006–2010		2011–2015		2016–2019		*trend p-value*
**N**	1975		2375		3265		
**Age, mean (SD)**	43.5 (18.9)		43.5 (17.9)		48.8 (19.3)		*<0.001*
	*f*	%	*f*	%	*f*	%	
**Female Sex**	500	25,3%	675	28,4%	905	27,7%	*0,57*
**Non-white**	800	40,5%	870	36,6%	1260	38,6%	*0,50*
**Private Insurance**	1250	63,6%	1430	60,3%	1710	52,8%	*0,001*
**Comorbidities**							
Hypertension	310	15,7%	600	25,3%	1075	32,9%	*<0.001*
Coronary Artery Disease	85	4,3%	85	3,6%	175	5,4%	*0,35*
Congestive Heart Failure	25	1,3%	30	1,3%	115	3,5%	*0,013*
Chronic Obstructive Pulmonary Disease	115	5,8%	180	7,6%	195	6,0%	*0,47*
Diabetes Mellitus	85	4,3%	195	8,2%	295	9,0%	*0,016*
Chronic Kidney Disease	20	1,0%	45	1,9%	90	2,8%	*0,15*
**Hospital Setting**							*<0.001*
Rural	60	3,1%	25	1,1%	15	0,5%	
Urban Nonteaching	175	9,1%	165	7,0%	140	4,3%	
Urban Teaching	1690	87,8%	2165	91,9%	3110	95,3%	
**Hospital Bedsize**							*0,44*
Small	25	1,3%	75	3,2%	85	2,6%	
Medium	310	16,1%	410	17,4%	565	17,3%	
Large	1590	82,6%	1870	79,4%	2615	80,1%	
**Patients admitted as Transfer**	220	11,5%	375	15,8%	575	17,7%	*0,027*
**Concomitant injury**							
Rib Fracture	1080	54,7%	1355	57,1%	2050	62,8%	*0,022*
Pneumothorax	365	18,5%	500	21,1%	700	21,4%	*0,49*
Hemothorax	215	10,9%	205	8,6%	375	11,5%	*0,29*
Hemopneumothorax	345	17,5%	475	20,0%	700	21,4%	*0,30*
Lung Injury	605	30,6%	705	29,7%	1230	37,7%	*0,008*
Kidney Injury	185	9,4%	300	12,6%	330	10,1%	*0,24*
Traumatic Brain Injury	850	43,0%	1590	66,9%	1210	37,1%	*<0.001*
Traumatic Cardiac Injury	85	4,3%	40	1,7%	55	1,7%	*0,013*
Pelvic Injury	35	1,8%	65	2,7%	75	2,3%	*0,64*
Skull Fracture	165	8,4%	310	13,1%	405	12,4%	*0,065*
Spinal Fracture without SCI	955	48,4%	1260	53,1%	1780	54,5%	*0,15*
Spinal Fracture with SCI	110	5,6%	100	4,2%	60	1,8%	*0,004*
**Concomitant Procedure**							
Intracranial Intervention	135	6,8%	250	10,5%	230	7,0%	*0,061*
Thoracic Intervention	995	50,4%	1315	55,4%	2190	67,1%	*<0.001*
Abdominal Intervention	1290	65,3%	1495	62,9%	600	18,4%	*<0.001*
							
**LOS - days**	18.8 (19.7)		16.6 (18.4)		15.0 (15.9)		*0,003*
**Cost - $**	241276.5 (216674.3)		331569.8 (350865.6)		348270.9 (360591.1)		*<0.001*
**Outcomes**							
Died	295	15,0%	335	14,1%	410	12,6%	*0,51*
Died within Hospital Day #1	130	6,6%	150	6,3%	140	4,3%	*0,19*
Discharge Home	770	39,0%	930	39,2%	1340	41,0%	*0,74*
**Complications**							
Paraplegia	25	1,3%	5	0,2%	100	3,1%	*<0.001*
Cardiac Complication	190	9,6%	410	17,3%	235	7,2%	*<0.001*
Pulmonary Complication	930	47,1%	1250	52,6%	1455	44,6%	*0,026*
Acute Renal Failure	150	7,6%	255	10,7%	700	21,4%	*<0.001*
Sepsis	80	4,1%	130	5,5%	160	4,9%	*0,62*

f = weighted number of patients; SCI = spinal cord ischemia; SD = standard deviation; LOS = length of stay; SD = standard deviation.

#### Concomitant injuries and interventions

Over the years, a statistically significant increasing trend in rib fractures (2006–2010 *vs*. 2016–2019; 55% *vs*. 63%; *p* trend = 0.022) and lung injuries (31% *vs*. 38%; *p* trend = 0.008) was seen in the whole BTAI cohort (Table 1). A decreasing trend was found for traumatic brain injury (43% *vs*. 37%; *p* trend <0.001), traumatic cardiac injury (4.3% *vs*. 1.7%; *p* trend = 0.013), and spinal fracture with spinal cord ischaemia (5.6% vs. 1.8%; *p* trend = 0.004). Evaluating the concomitant procedures, statistically significantly more patients had additional thoracic interventions besides TEVAR (50% *vs*. 67%; *p* trend <0.001), and statistically significantly fewer patients had additional abdominal interventions over the years (65% *vs*. 18%; *p* trend <0.001).

#### Outcomes in the overall cohort

The data demonstrated no statistically significant up or downward trend in in hospital mortality in the overall BTAI cohort (2006–2010 *vs*. 2016–2019; 15% *vs*. 12.6%; *p* trend = 0.51) (Table 1 and Fig. 2). Otherwise, a significant upward trend in paraplegia (1.3% *vs*. 3.1%; *p* trend <0.001) and a downward trend for cardiac complications (9.6% *vs*. 7.2%; *p* trend <0.001) and pulmonary complications (47% *vs*. 45%; *p* trend = 0.026) was found.

### Non-operative management cohort

#### Patient characteristics

In the NOM cohort, the average age statistically significantly increased (2006–2010 *vs*. 2016–2019; 45 years *vs*. 50 years; *p* trend <0.001) (Table 2). Over the years, a statistically significant increasing trend in patients treated in an urban teaching hospital (87% *vs*. 94%; *p* trend <0.001) was found. Also, more patients were admitted as transfers from another hospital (8.9% *vs*. 18%; *p* trend = 0.002).

**Table 2:** Demographics, comorbidities, hospital characteristics, concomitant injuries, interventions and outcomes in BTAI patients treated with nonoperative management over time.

	Nonoperative Management						
	2006–2010		2011–2015		2016–2019		*trend p-value*
**N**	1115		1335		1980		
**Age, mean (SD)**	45.3 (20.0)		43.9 (17.7)		50.2 (19.5)		*<0.001*
	*f*	%	*f*	%	*f*	%	
**Female Sex**	315	28,3%	410	30,7%	585	29,5%	*0,35*
**Non-white**	430	38,6%	500	37,5%	785	39,6%	*0,89*
**Private Insurance**	680	61,3%	580	43,6%	910	46,2%	*0,53*
**Comorbidities**							
Hypertension	185	16,6%	335	25,1%	660	33,3%	*<0.001*
Coronary Artery Disease	45	4,0%	30	2,2%	125	6,3%	*0,055*
Congestive Heart Failure	20	1,8%	15	1,1%	70	3,5%	*0,034*
Chronic Obstructive Pulmonary Disease	55	4,9%	120	9,0%	135	6,8%	*0,45*
Diabetes Mellitus	65	5,8%	115	8,6%	200	10,1%	*0,028*
Chronic Kidney Disease	10	0,9%	20	1,5%	55	2,8%	*0,030*
**Hospital Setting**							*<0.001*
Rural	30	2,7%	20	1,5%	5	0,3%	
Urban Nonteaching	115	10,4%	115	8,7%	110	5,6%	
Urban Teaching	960	86,9%	1190	89,8%	1865	94,1%	
**Hospital Bedsize**							*0,068*
Small	15	1,4%	55	4,2%	70	3,5%	
Medium	175	15,8%	210	15,8%	385	19,4%	
Large	915	82,8%	1060	80,0%	1525	77,0%	
**Patients admitted as Transfer**	95	8,9%	200	15,0%	365	18,4%	*0,002*
**Concomitant Injury**							
Rib Fracture	575	51,6%	750	56,2%	1215	61,4%	*<0.001*
Pneumothorax	175	15,7%	325	24,3%	425	21,5%	*0,041*
Hemothorax	120	10,8%	90	6,7%	215	10,9%	*0,62*
Hemopneumothorax	210	18,8%	255	19,1%	410	20,7%	*0,43*
Lung Injury	315	28,3%	410	30,7%	730	36,9%	*0,003*
Kidney Injury	80	7,2%	200	15,0%	210	10,6%	*0,14*
Traumatic Brain Injury	460	41,3%	885	66,3%	720	36,4%	*0,22*
Traumatic Cardiac Injury	50	4,5%	30	2,2%	50	2,5%	*0,44*
Pelvic Injury	15	1,3%	35	2,6%	60	3,0%	*0,23*
Skull Fracture	95	8,5%	170	12,7%	225	11,4%	*0,14*
Spinal Fracture without SCI	550	49,3%	730	54,7%	1110	56,1%	*0,013*
Spinal Fracture with SCI	75	6,7%	50	3,7%	45	2,3%	*0,009*
**Concomitant Procedure**							
Intracranial Intervention	55	4,9%	65	4,9%	105	5,3%	*0,77*
Thoracic Intervention	585	52,5%	725	54,3%	905	45,7%	*0,097*
Abdominal Intervention	670	60,1%	795	59,6%	310	15,7%	*<0.001*
**LOS – days**	17.2 (20.6)		13.8 (17.5)		13.2 (14.8)		*0,056*
**Cost - $**	191980 (198482)		259767 (312732)		281383 (330705)		*<0.001*
**Outcomes**							
Died	235	21,1%	265	19,9%	295	14,9%	*0,034*
Died within Hospital Day #1	115	10,3%	135	10,1%	120	6,1%	*0,006*
Discharge Home	390	35,0%	555	41,6%	835	42,2%	*0,079*
**Complications**							
Paraplegia	15	1,3%	5	0,4%	65	3,3%	*0,014*
Cardiac complication	115	10,3%	80	6,0%	140	7,1%	*0,11*
Pulmonary Complication	15	1,3%	80	6,0%	740	37,4%	*0,055*
Postop Acute Renal Failure	100	9,0%	105	7,9%	405	20,5%	*<0.001*
Postop Sepsis	70	6,3%	65	4,9%	95	4,8%	*0,87*

f = weighted number of patients; SCI = spinal cord ischemia; LOS = length of stay; SD = standard deviation.

#### Concomitant injuries and interventions

Over time, patients in the NOM cohort more frequently had concomitant injuries, including rib fractures (2006–2010 *vs*. 2016–2019; 52% *vs*. 61%; *p* trend <0.001), pneumothorax (16% *vs*. 22%; *p* trend = 0.041), lung injuries (28% *vs*. 37%; *p* trend = 0.003), and spinal fractures without SCI (49% *vs.* 56%; *p* trend = 0.013) (Table 2). There was a decrease in patients with spinal fractures with SCI (6.7% *vs*. 2.3%; *p* trend = 0.009). Regarding the concomitant procedures, statistically significantly fewer patients had an abdominal intervention in each time period (60% *vs*. 16%; *p* trend <0.001).

#### Outcomes after non-operative management

The yearly mortality rates fluctuated (Fig. 2 and Supplementary Table S1). The trend analysis per time group demonstrated a decrease in the overall mortality after multivariable logistic regression (2006–2010 *vs*. 2016–2019; 21% *vs*. 15%; *p* trend = 0.034) (Table 2). Patients who died within one day (10% *vs*. 6.1%; *p* trend = 0.006) decreased over time.

### TEVAR cohort

#### Patient characteristics

In all BTAI patients treated with TEVAR, the average age significantly increased (2006–2010 *vs*. 2016–2019; 41years *vs*. 46 years; *p* trend <0.001) (Table 3). No other differences in patient demographics were found. An increasing trend was seen in patients treated in an urban teaching hospital (91% *vs*. 97%; *p* trend = 0.048). Furthermore, most of the patients were treated in a large hospital but there was no significant difference in hospital transfers in the patients treated with TEVAR over the years.

#### Concomitant injuries and interventions

Evaluating the concomitant injuries in the TEVAR cohort, a statistically significant decreasing trend in patients with TBI (2006–2010 *vs*. 2016–2019; 47% *vs*. 39%; *p* trend = 0.007), traumatic cardiac injury (4.4% *vs*. 0%; *p* trend <0.001), and spinal fractures with SCI (5.2% *vs*. 1.2%; *p* trend = 0.011) was found over the years (Table 3). There was a statistically significant decrease in patients who had an abdominal intervention (72% *vs*. 22%; *p* trend <0.001). Evaluating the timing of TEVAR, an increase in patients treated with TEVAR within 24 hours after admission was found (65% *vs*. 83%; *p* trend <0.001).

**Table 3:** Demographics, comorbidities, and hospital characteristics in BTAI patients treated with thoracic endovascular aortic repair over time.

		TEVAR						
		2006–2010		2011–2015		2016–2019		*trend p-value*
**N**		675		965		1240		
**Age, mean (SD)**		41.1 (16.9)		43.2 (18.0)		46.4 (18.9)		*<0.001*
		*f*	%	*f*	%	*f*	%	
**Female Sex**		120	17,8%	250	25,9%	295	23,8%	*0,24*
**Non-white**		305	45,2%	350	36,3%	460	37,1%	*0,13*
**Private Insurance**		450	67,2%	510	52,8%	520	42,1%	*0,028*
**Comorbidities**								
Hypertension		100	14,8%	240	24,9%	390	31,5%	*<0.001*
Coronary Artery Disease		35	5,2%	50	5,2%	45	3,6%	*0,45*
Congestive Heart Failure		5	0,7%	10	1,0%	40	3,2%	*0,061*
Chronic Obstructive Pulmonary Disease		55	8,1%	50	5,2%	60	4,8%	*0,23*
Diabetes Mellitus		20	3,0%	75	7,8%	95	7,7%	*0,10*
Chronic Kidney Disease		5	0,7%	20	2,1%	30	2,4%	*0,27*
**Hospital Setting**								*0,048*
Rural		25	3,9%	5	0,5%	5	0,4%	
Urban Nonteaching		30	4,7%	35	3,7%	30	2,4%	
Urban Teaching		580	91,3%	915	95,8%	1205	97,2%	
**Hospital Bedsize**								*0,22*
Small		5	0,8%	20	2,1%	15	1,2%	
Medium		115	18,1%	200	20,9%	165	13,3%	
Large		515	81,1%	735	77,0%	560	85,5%	
**Patients admitted as Transfer**		110	16,4%	155	16,1%	205	16,7%	*0,96*
**Concomitant injury**								
Rib Fracture		405	60,0%	575	59,6%	805	64,9%	*0,27*
Pneumothorax		150	22,2%	165	17,1%	270	21,8%	*0,84*
Hemothorax		85	12,6%	105	10,9%	155	12,5%	*0,90*
Hemopneumothorax		110	16,3%	210	21,8%	280	22,6%	*0,21*
Lung Injury			37,0%	265	27,5%	480	38,7%	*0,47*
Kidney Injury		95	14,1%	90	9,3%	120	9,7%	*0,25*
Traumatic Brain Injury		320	47,4%	665	68,9%	485	39,1%	*0,007*
Traumatic Cardiac Injury		30	4,4%	10	1,0%	0	0,0%	*<0.001*
Pelvic Injury		15	2,2%	30	3,1%	15	1,2%	*0,40*
Skull Fracture		55	8,1%	135	14,0%	170	13,7%	*0,15*
Spinal Fracture without SCI		340	50,4%	515	53,4%	650	52,4%	*0,66*
Spinal Fracture with SCI		35	5,2%	45	4,7%	15	1,2%	*0,011*
**Concomitant Procedure**								
Intracranial Intervention		75	11,1%	175	18,1%	120	9,7%	*0,32*
Thoracic Intervention		305	45,2%	540	56,0%	1240	100,0%	*<0.001*
Abdominal Intervention		490	72,6%	645	66,8%	275	22,2%	*<0.001*
**Time till TEVAR ≤24 hours**		440	65,2%	720	74,6%	1035	83,5%	*<0.001*
**LOS - days**		20.7 (17.3)		20.7 (19.3)		17.5 (17.2)		*0,010*
**Cost - $**		296769 (219485)		430762 (384259)		434284 (339922)		*<0.001*
**Outcomes**								
Died		45	6,7%	45	4,7%	110	8,9%	*0,41*
Died within Hospital Day #1		5	0,7%	10	1,0%	20	1,6%	*0,43*
Discharge Home		290	43,0%	355	36,8%	500	40,3%	*0,83*
**Postoperative complications**								
Paraplegia		5	0,7%	0	0,0%	35	2,8%	*0,045*
Cardiac Complication		65	9,6%	185	19,2%	85	6,9%	*0,086*
Pulmonary Complication		20	2,9%	65	6,7%	685	55,2%	*0,41*
Acute Renal Failure		50	7,4%	135	14,0%	275	22,2%	*<0.001*
Sepsis		10	1,5%	65	6,7%	65	5,2%	*0,17*

f = weighted number of patients; SCI = spinal cord ischemia; SD = standard deviation; TEVAR = thoracic endovascular aortic repair.

#### Post-operative outcomes

The yearly mortality rate fluctuated (Fig. 2 and Supplementary Table S1), but the overall mortality (2006–2010 *vs*. 2016–2019; 6.7% *vs*. 8.9%; *p* trend = 0.41) and death within one day after arrival (0.7% *vs*. 1.6%; *p* trend = 0.43) remained similar over the three time periods (Table 3). There was a rise in patients with paraplegia over the years (0.7% *vs*. 2.8%; *p* trend = 0.045). Furthermore, there was a downward trend in the length of hospital stay (21 days [SD 18] *vs*. 18 days [SD 17]; *p* tend = 0.010).

### Open aortic repair cohort

#### Patient characteristics

In all patients with BTAI treated with OAR, the median age remained similar in all three time periods (Table 4). Most patients were treated in an urban teaching hospital, without significant trends over the years. Most patients were treated in a large hospital, and there was no difference in hospital transfers over the years.

**Table 4:** Demographics, comorbidities, hospital characteristics, concomitant injuries, interventions and outcomes in BTAI patients treated with open aortic repair over time.

	OAR						
	2006–2010		2011–2015		2016–2019		*trend p-value*
**N**	185		75		45		
**Age, mean (SD)**	38.3 (17.4)		40.1 (19.0)		52.2 (16.3)		*0,092*
	*f*	%	*f*	%	*f*	%	
**Female Sex**	65	35,1%	15	20,0%	25	56,0%	*0,33*
**Non-white**	65	35,1%	20	27,0%	15	33,0%	*0,39*
**Private Insurance**	120	64,9%	20	27,0%	29	44,0%	*0,45*
**Comorbidities**							
Hypertension	25	13,5%	25	33,0%	25	56,0%	*<0.001*
Coronary Artery Disease	5	2,7%	5	7,0%	5	11,0%	*0,27*
Congestive Heart Failure	0	0,0%	5	7,0%	5	11,0%	*0,019*
Chronic Obstructive Pulmonary Disease	5	2,7%	10	13,0%	0	0,0%	*0,74*
Diabetes Mellitus	0	0,0%	5	7,0%	0	0,0%	*0,36*
Chronic Kidney Disease	5	2,7%	5	7,0%	5	11,0%	*0,11*
**Hospital Setting**							*0,56*
Rural		2,7%	0	0,0%	5	11,0%	
Urban Nonteaching	30	16,2%	15	20,0%	0	0,0%	
Urban Teaching	150	81,1%	60	80,0%	40	89,0%	
**Hospital Bedsize**							*0,17*
Small	5	2,7%	0	0,0%	0	0,0%	
Medium	20	10,8%	0	0,0%	15	33,0%	
Large	160	86,5%	75	100,0%	30	67,0%	
**Patients admitted as Transfer**	15	8,3%	20	27,0%	5	12,0%	*0,091*
**Concomitant injury**							
Rib Fracture	100	54,1%	30	40,0%	30	67,0%	*0,72*
Pneumothorax	40	21,6%	10	13,0%	5	11,0%	*0,51*
Hemothorax	10	5,4%	10	13,0%	5	11,0%	*0,74*
Hemopneumothorax	25	13,5%	10	13,0%	10	22,0%	*0,55*
Lung Injury	40	21,6%	30	40,0%	20	44,0%	*0,18*
Kidney Injury	10	5,4%	10	13,0%	0	0,0%	*0,66*
Traumatic Brain Injury	70	37,8%	40	53,0%	5	11,0%	*0,46*
Traumatic Cardiac Injury	5	2,7%	0	0,0%	5	11,0%	*0,20*
Pelvic Injury	5	2,7%	0	0,0%	0	0,0%	*0,40*
Skull Fracture	15	8,1%	5	7,0%	10	22,0%	*0,10*
Spinal Fracture without SCI	65	35,1%	15	20,0%	20	44,0%	*0,79*
Spinal Fracture with SCI	0	0,0%	5	7,0%	0	0,0%	*0,88*
**Concomitant Procedure**							
Intracranial Intervention	5	2,7%	10	13,0%	5	11,0%	*0,064*
Thoracic Intervention	105	56,8%	50	67,0%	45	100,0%	*0,012*
Abdominal Intervention	130	70,3%	55	73,0%	15	33,0%	*0,15*
							
**LOS - days**	21.4 (22.0)		12.8 (8.5)		21.4 (20.1)		*0,18*
**Cost - $**	220698 (152045)		332246 (226839)		861722 (930624)		*0,024*
**Outcomes**							
Died	15	8,1%	25	33,0%	5	11,0%	*0,12*
Died within Hospital Day #1	10	5,4%	5	7,0%	0	0,0%	*0,88*
Discharge Home	90	48,6%	20	27,0%	5	11,0%	*0,050*
**Postoperative complications**							
Paraplegia	5	2,7%	0	0,0%	0	0,0%	*0,56*
Cardiac Complication	10	5,4%	20	27,0%	10	22,0%	*0,042*
Pulmonary Complication	145	48,0%	35	47,0%	30	67,0%	*0,42*
Acute Renal Failure	0	0,0%	15	20,0%	20	44,0%	*<0.001*
Sepsis	0	0,0%	0	0,0%	0	0,0%	*0,56*

f = weighted number of patients; SCI = spinal cord ischemia; SD = standard deviation; LOS = length of stay; SD = standard deviation.

#### Concomitant injuries and interventions

No up or downward trend in concomitant injuries was found in the OAR cohort (Table 4).

##### Post-operative outcomes

Over the years, the overall mortality (2006–2010 *vs*. 2016–2019; 8.0% *vs*. 11%; *p* trend = 0.12) and the deaths within one day after arrival (3.0% *vs*. 0.0%; *p* trend = 0.88) remained similar (Table 4). Furthermore, a downward trend was found in patients discharged home (49% *vs*. 11%; *p* trend = 0.050).

### Factors associated with mortality after BTAI

#### Non-operative management cohort

It was found that after adjustments, there was no significant difference in mortality across the three time periods (Table 5). Concomitant injuries to the lung and kidney were associated with lower mortality (aOR 0.61, *p* = 0.010; aOR 0.34, *p* < 0.010, respectively), while haemothorax and spinal fracture without SCI were associated with increased mortality (aOR 3.1, *p* < 0.010; aOR 1.7, *p* < 0.010, respectively). As for procedures performed during the index hospitalisation, non-operatively managed patients who also underwent thoracic intervention, including procedures to the heart and the lungs, had a higher mortality rate (aOR 2.2, *p* < 0.010).

**Table 5:** Multivariable predictors of mortality after blunt thoracic aortic injury.

Predictors	Non-operative management			TEVAR		
	aOR	95% CI	*p* value	aOR	95% CI	*p* value
*Year range*						
2006–2010	ref	–	–	ref	–	–
2011–2015	0.94	0.61–1.47	0.80	0.41	0.15–1.13	0.090
2016–2019	0.65	0.42–1.02	0.060	0.97	0.33–2.88	0.96
*Adjusters*						
Female	0.85	0.57–1.25	0.40	0.46	0.17–1.25	0.13
Non-white	0.85	0.59–1.23	0.40	0.82	0.39–1.71	0.59
Private insurer	1.62	1.13–2.33	0.010	0.86	0.43–1.70	0.66
Time to TEVAR	–	–	–	0.51	0.20–1.32	0.17
*Concomitant injuries*						
Brain injury	1.34	0.94–1.93	0.11	2.94	1.38–6.33	<0.010
Cardiac injury	2.40	0.98–5.85	0.050	2.18	0.22–21.95	0.51
Lung injury	0.61	0.41–0.80	0.010	0.94	0.45–1.95	0.86
Rib fracture	0.81	0.56–1.16	0.25	1.24	0.60–2.61	0.56
Pneumothorax	1.12	0.70–1.80	0.65	0.85	0.30–2.41	0.76
Haemothorax	3,08	1.77–5.34	<0.010	2.25	0.83–6.05	0.11
Haemopneumothorax	1,55	0.99–2.44	0.06	1.87	0.81–4.31	0.14
Kidney injury	0.34	0.16–0.70	<0.010	0.17	0.02–1.29	0.090
Pelvic injury	1.10	0.37–3.24	0.86	–	–	–
Vertebral fractures with SCI	1.38	0.63–3.05	0.43	2.28	0.53–9.88	0.27
Vertebral fractures without SCI	1.73	1.20–2.48	<0.010	1.18	0.59–2.36	0.64
*Additional procedures*						
Intracranial intervention	1.13	0.54–2.41	0.75	3.11	1.39–6.97	<0.010
Thoracic intervention	2.21	1.50–3.24	<0.010	1.69	0.58–4.91	0.33
Abdominal intervention	0.75	0.51–1.12	0.16	0.95	0.43–2.12	0.90

CI = confidence interval; SCI = spinal cord ischaemia.

#### TEVAR cohort

In the patients treated with TEVAR, it was found that after adjustments, there was no significant difference in mortality across the three time periods (Table 5). Only TBI was associated with increased mortality (aOR 2.9, *p* < 0.010), while all other comorbidities and concomitant injuries had no significant effect. Notably, BTAI patients managed with TEVAR who also underwent intracranial intervention had a higher mortality rate (aOR 3.1, *p* < 0.010).

## DISCUSSION

Over the study period of 14 years, the estimated number of BTAI in trauma patients admitted to the hospital in the US increased from 375 patients in 2006 to 750 patients in 2019. Interestingly, Tomas and colleagues found a significant decrease in motor vehicle transport (MVT) occupants, MVT motorcyclists, and other blunt trauma in the US over the years.^20^ The rise in patients with BTAI may be attributed to higher survival rates of motor vehicle crashes by improvements in prevention measures, such as obligatory seatbelts, airbags, and crash avoidance technologies, as well as improvements in pre-hospital and hospital care, including the implementation of advanced trauma life support and haemorrhage management with the massive transfusion protocol.^4^^,^^10^^,^^11^^,^^21^, ^22^, ^23^, ^24^ Furthermore, the widespread adoption of CTA as the preferred imaging modality has likely enhanced the detection of smaller aortic injuries.^4^^,^^10^^,^^11^ However, prior research indicates that the most substantial increase in CTA utilisation occurred between 1994 and 2007.^9^^,^^25^ Additionally, Malhotra *et al.* observed an increase in the detection of minimal aortic injuries using CT scans in their study between 1994 and 2000 evaluating patients with BTAI.^11^ These findings suggest that significant advancements in CT technology, including improved resolution and faster acquisition times, were already in progress before the timeframe of the current study. While ongoing improvement in imaging technology may have had a modest impact on the findings, it is unlikely to be the primary driver. Despite the doubling of BTAI cases over the years, the mortality rate within the entire BTAI cohort has remained relatively stable over the years, underscoring the complexity of this condition and the multifaceted challenges in its management.

With the increase in patients presenting with BTAI, the utilisation of TEVAR significantly increased, largely replacing OAR. Ultee and colleagues also demonstrated an increase in the use of TEVAR from 1% in 2005 to 40% in 2011 in all patients treated for BTAI, utilising the NIS database.^14^ The rise in the use of TEVAR can be explained by the FDA approval of TEVAR for aneurysm repair in 2005, resulting in the off label use of TEVAR for patients with BTAI.^13^^,^^26^ In the following years, several studies were conducted comparing TEVAR and OAR.^6^^,^^9^^,^^27^, ^28^, ^29^ In 2008, Demetriades and colleagues found in the AAST trial that TEVAR in patients with BTAI was associated with fewer blood transfusions and lower mortality compared with OAR.^9^ Rousseau *et al.* also found TEVAR to be a valuable alternative for OAR in patients with BTAI.^29^ Surgeons also seem to prefer TEVAR over OAR for young and low risk patients without major associated injuries.^9^ In 2012, the FDA approved the Gore TAG thoracic endoprosthesis and the Medtronic Valiant Thoracic Stent Graft for the treatment of patients with BTAI, which contributed to the improvement and availability of endograft devices in patients with BTAI.^30^^,^^31^

The yearly mortality rate after TEVAR in patients with BTAI fluctuated, there was no significant up or downward trend in in hospital mortality rates over the years. This suggests that there is still potential for improvement in treating an aortic injury with TEVAR. An important aspect in the endovascular treatment is timing. The current results show that most patients were treated with TEVAR within the first 24 hours after hospital arrival, and this number has increased over the years. However, the research group recently found that delayed TEVAR (>24 hours after admission) was associated with a lower mortality rate in patients with BTAI compared with early TEVAR (≤24 hours after admission).^32^ This was also found in the study by Marcaccio *et al.*.^33^ A possible reason for the higher number of patients treated within 24 hours after admission can be the current SVS guidelines, which recommend aortic repair within 24 hours once other more life threatening injuries are treated.^13^ However, the European Society of Vascular Surgery (ESVS) recently published new guidelines for BTAI and they suggested that delayed TEVAR (>24 hours) should be considered in patients with BTAI.^34^ Therefore, there is potential for improvement in this area in the future. Optimal timing of TEVAR is highly relevant and warrants further investigation. However, the NIS database is not the right database to use for this specific question, as more granular clinical data would be required, such as the severity of aortic injury and the overall trauma burden.

Interestingly, the number of patients with BTAI who were conservatively treated remained similar, as was the mortality rate over the years, despite an increase in age, comorbidities, and concomitant injuries over time. A notable finding is the decreasing trend in mortality within one day. The NOM cohort consists of patients lacking ICD codes for TEVAR or OAR, resulting in patients defined as NOM even if they died before the possibility of receiving any treatment. The current results insinuate that patients were chosen to be conservatively treated, which resulted in lower mortality rates. De Mestral *et al.* also found a decrease in mortality rate in patients treated with NOM and suggested that conservative treatment should be of more focus in the current treatment strategy.^35^ Moreover, many of the conservatively treated patients were treated in large bed number and urban teaching hospitals, mainly level 1 trauma centers.^14^ But also the increasing trend in hospital transfer, likely to level 1 trauma centres, might also contribute to the decreased mortality rate. Prior research evaluating the effect of the transfer of severely injured patients on mortality found significantly lower 24 hour and 30 day mortality rates if patients were transferred to high level trauma centers.^36^, ^37^, ^38^ The inter-hospital transfer was mainly beneficial for patients with traumatic brain injury or critical injuries (ISS ≥25). A recent systematic review and meta-analysis evaluating the association between level of trauma care and the outcomes in severely injured patients found that patients treated in a level 1 trauma centre have survival benefits.^39^ An organised trauma system is of importance with the increasing incidence of patients with BTAI, since ensuring the most appropriate level of trauma care in the least amount of time might reduce mortality in this population.

In this current endovascular era where TEVAR is the standard of treatment for patients with BTAI, there appears to be room for further improvement in both operative treatment and conservative management. The current results show an association between mortality and TBI in patients treated with TEVAR. Research shows that 20–50% of the patients with BTAI also suffer from TBI, corresponding to the numbers found in this study.^25^^,^^40^ Patients with TBI need adequate perfusion of the brain, requiring good systolic blood pressure and oxygenation, but anticoagulation therapy should be avoided.^41^^,^^42^ Sometimes, vasoactive haemodynamic support is needed, which makes conservative management of the aortic injury unwise. On the other hand, operative repair of the aortic injury can cause hypoxaemia and hypotension, contributing to a secondary brain injury.^43^ So, both NOM and TEVAR have challenges in patients with BTAI and TBI. Interestingly, Rabin *et al.* showed that early TEVAR (<24 hours after admission) for BTAI was associated with progressive TBI and increased aortic morbidity and mortality.^41^ Also, Zambetti and colleagues found mortality benefits in patients with TBI and BTAI treated with delayed TEVAR (more than nine hours after admission).^44^ As previously discussed, the timing of TEVAR is of great importance. The recent guidelines by the ESVS recommended delayed TEVAR in patients with concomitant TBI, allowing for initial neurological evaluation and stabilisation of cerebral perfusion before subjecting the patient to the potential risk of endovascular repair.^34^ The guidelines further emphasise that, in such patients, a blood pressure management strategy should aim for systolic values >100 mmHg to support cerebral autoregulation, while also avoiding hypertensive peaks that may aggravate the aortic injury. Multidisciplinary coordination between trauma, vascular surgery, and neurocritical care teams is therefore essential to individualise timing and optimise outcomes.

This study must be interpreted in the context of its retrospective study design. Data up to the year 2019 were included. Although more recent data would have been preferable, the 2019 cutoff still allows for meaningful and relevant analysis. The trends and associations observed in this dataset remain valuable for understanding the management and outcomes of BTAI, particularly give the relatively stable treatment protocols in recent years. Due to the long period of inclusion and improvement of diagnostic tools over the years, there is a chance of classification bias. The transition from ICD-9 to ICD-10 coding contributed to this, which might have resulted in missed diagnoses in the earlier years. Although it was attempted to account for possible influencing factors in the statistical analysis, there might have been some unmeasured confounding due to limitations in the NIS database. Likely, there was bias related to coding and billing practices. Over time, in the US, a lot has changed regarding diagnosis related codes. The alteration in coding introduces a distorted representation of the rise in the number of patients with specific comorbidities and complications. This might be the case for paraplegia, and will probably also explain the extremely high rates of additional thoracic interventions. The NIS database does not include ISS and AIS scores. Instead, the presence of specific injuries was used by using ICD codes as a proxy to account for injury patterns and their potential impact on the outcomes. It is recognised that this simplification may limit the interpretability of the findings. Other limitations that should be considered are missing injury related data, such as aortic injury grading, injury severity scores, and aortic related information. Furthermore, it was not possible to evaluate cause and effect, so complications could not be related to a certain injury or the treatment received. However, from prior research, it is known that the improvement of endografts over the years has led to a decrease in procedure related complications.^45^ The analysis in the OAR cohort was limited by a small patient population. Unfortunately, the NOM cohort lacked specific ICD codes, and the variables that would have contributed to deciding between NOM and palliative care, such as injury severity score and aortic injury grade, were unknown, potentially impacting mortality rates. Evaluating long term follow up is beyond the scope of the NIS database.

### CONCLUSION

This trend analysis demonstrated an increase in the incidence of BTAI over the past 14 years. The use of TEVAR increased, largely replacing OAR, while in hospital mortality remained significant. BTAI is a complex condition with multifaceted challenges in its management and there is still room for improvement in the treatment with TEVAR and NOM for patients in the coming years. An organised trauma system is important to contribute to improved outcomes in the future.

## FUNDING

None.

## Conflict of interests

HV is a consultant of Medtronic, WL Gore, Terumo, Endologix, Philips.
