# Supplementary material for: Trends in Management and Outcomes of Blunt Thoracic Aortic Injury in the United States
Source: EJVES Vasc Forum. 2025 Jul 21;64:128–40. doi: 10.1016/j.ejvsvf.2025.07.004 (PMC12547014; doi:10.1016/j.ejvsvf.2025.07.004)
Supplement: Multimedia component 1 [file mmc1.pdf]

Supplementary Table S1. Presentation of patients with blunt thoracic aortic injury over the years and the number who died.

	2006	***2007***	***2008***	***2009***	***2010***	***2011***	***2012***	***2013***	***2014***	***2015***	***2016***	***2017***	***2018***	***2019***
Total number of patients	375	410	425	275	490	485	420	570	500	400	800	805	910	750
OAR:	60 (16%)	35 (8.5%)	50 (11.8%)	20 (7.3%)	20 (4.0%)	40 (8.3%)	25 (6.0%)	5 (0.9%)	5 (1.0%)	5 (1.2%)	10 (1.2%)	10 (1.2%)	20 (2.2%)	10 (1.3%)
Dead:	0 (0%)	5 (14%)	5 (10%)	0 (0%)	5 (25%)	5 (13%)	10 (40%)	5 (100%)	5 (100%)	0 (0%)	5 (50%)	0 (0%)	0 (0%)	0 (0%)
TEVAR:	65 (17.3%)	80 (19.5%)	170 (40.0%)	125 (45.5%)	235 (48.0%)	185 (38.1%)	155 (36.9%)	235 (41.2%)	195 (39.0%)	195 (48.8%)	280 (35.0%)	305 (37.9%)	375 (41.2%)	280 (37.3%)
Dead:	10 (15%)	5 (6.3%)	0 (%)	5 (4%)	25 (11%)	10 (5.4%)	20 (13%)	0 (0%)	5 (2.6%)	10 (5.1%)	30 (11%)	20 (6.6%)	45 (12%)	15 (5.4%)
Nonoperative:	250 (66.7%)	295 (72.0%)	205 (48.2%)	130 (57.2%)	235 (48.0%)	260 (53.6%)	240 (57.1%)	330 (57.9%)	300 (60.0%)	200 (50%)	510 (63.8%)	490 (60.9%)	515 (56.6%)	460 (61.4%)
Dead:	55 (22%)	50 (17%)	30 (15%)	40 (31%)	60 (26%)	35 (13%)	70 (29%)	55 (17%)	60 (20%)	45 (23%)	55 (11%)	110 (22%)	45 (8.7%)	85 (18%)

OAR: open aortic repair, TEVAR: thoracic endovascular aortic repair
